# Selective Iron(III) ion uptake using CuO-TiO_2_ nanostructure by inductively coupled plasma-optical emission spectrometry

**DOI:** 10.1186/1752-153X-6-158

**Published:** 2012-12-17

**Authors:** Mohammed M Rahman, Sher Bahadar Khan, Hadi M Marwani, Abdullah M Asiri, Khalid A Alamry

**Affiliations:** 1 Center of Excellence for Advanced Materials Research (CEAMR), King Abdulaziz University, P.O. Box 80203, Jeddah, 21589, KSA; 2 Chemistry Department, Faculty of Science, King Abdulaziz University, P.O. Box 80203, Jeddah, 21589, KSA

**Keywords:** CuO-TiO_2_ nanosheets, Wet-chemical process, Optical property, Structural property, Adsorption isotherm, Iron(III) ion detection

## Abstract

**Background:**

CuO-TiO_2_ nanosheets (NSs), a kind of nanomaterials is one of the most attracting class of transition doped semiconductor materials due to its interesting and important optical, electrical, and structural properties and has many technical applications, such as in metal ions detection, photocatalysis, Chemi-sensors, bio-sensors, solar cells and so on. In this paper the synthesis of CuO-TiO_2_ nanosheets by the wet-chemically technique is reported.

**Methods:**

CuO-TiO_2_ NSs were prepared by a wet-chemical process using reducing agents in alkaline medium and characterized by UV/vis., FT-IR spectroscopy, X-ray photoelectron spectroscopy (XPS), powder X-ray diffraction (XRD), and field-emission scanning electron microscopy (FE-SEM) etc.

**Results:**

The structural and optical evaluation of synthesized NSs were measured by XRD pattern, Fourier transform infrared (FT-IR) and UV–vis spectroscopy, respectively which confirmed that the obtained NSs are well-crystalline CuO-TiO_2_ and possessing good optical properties. The morphological analysis of CuO-TiO_2_ NSs was executed by FE-SEM, which confirmed that the doped products were sheet-shaped and growth in large quantity. Here, the analytical efficiency of the NSs was applied for a selective adsorption of iron(III) ion prior to detection by inductively coupled plasma-optical emission spectrometry (ICP-OES). The selectivity of NSs towards various metal ions, including Au(III), Cd(II), Co(II), Cr(III), Fe(III), Pd(II), and Zn(II) was analyzed.

**Conclusions:**

Based on the selectivity study, it was confirmed that the selectivity of doped NSs phase was the most towards Fe(III) ion. The static adsorption capacity for Fe(III) was calculated to be 110.06 mgg^−1^. Results from adsorption isotherm also verified that the adsorption process was mainly monolayer-adsorption onto a surface containing a finite number of CuO-TiO_2_ NSs adsorption sites.

## Introduction

Transition-metal doped semiconductor nanomaterial have attracted significant attention due to their potential applications in fabrication of nano-scale electronics, electro-analytical, selective metal-ions detection, opto-electronics, biological devices, electron field-emission sources for emission displays, bio-chemical detections, surface-enhanced Raman properties, and immense-potential applications etc. [1,2]. It is exhibited a regular morphological nanostructure, which composed a number of regular phases with geometrically-coordinated metals and oxide atoms along the axes. Doped materials have also concerned significant research effort for their exceptional and outstanding properties as well as versatile applications [3,4]. In last decade, nano-sized transition-metal oxides have been widely studied as promising anodes for Laser-Induced Break-down Spectroscopy’s since they were first reported by Tarascon et al. [5,6]. Among them, undoped copper oxide (CuO) has attracted much interest owing to their high theoretical capacity, highly-stable, non-toxic, economical approach, and facile synthesis. It is a p-type semiconductor material with a band-gap energy [7-9], which is studied for various applications in bio-materials [10], photo-conductive [11] electro-magnetic [12], and super-conductors mico-devices [13] etc. Various efforts have been focused toward the fabrication of nano-structured CuO to improve their performance in currently existing applications, which is considered as one of the promising artificial mediators owing to their properties and functionalities [14-21]. However, it is still a big challenge to attain high-rate capability and crystallinity of copper oxide doped semiconductor nanomaterials. It is well accepted that a smaller size of CuO can lead to higher capacity and higher surface capability. This reduces the over potential and allows faster reaction kinetics to detect the metal ions [22-24]. Therefore, the synthesis of low-dimensional CuO nanostructures is a promising approach to make a significant improvement of large-surface area and high-aspect-ratio in doped nanostructures. Due to the significant properties of semiconductors, the doped nanomaterials were attained a considerable attention in terms of controlled growth of crystalline materials in huge quantity. Various growth mechanism have been employed, including the vapor–liquid–solid growth [25], thin-film growth [26], vapor-solid growth [27], wet-chemical methods [28], and electro-spinning [29] etc. Titanium dioxide is the promising host-material as semiconductor having high photo-chemical stability and large-surface area with low economical-cost. Well-dispersed undoped titania dioxide nanostructure materials with very fine sizes are promising in many significant applications such as pigments, adsorbents and catalytic supports [30-32]. In almost all of these cases, when the dimension of nanomaterials is reduced significantly, especially to several nano-meter scales, some novel optical, morphological, and structural properties are expected, owing to the large surface-to-volume ratio [33]. In addition, the development of simple, rapid and efficient techniques has achieved a huge interest for monitoring metal ions in the environment. Several analytical methods have been applied to analyze metal ions in aqueous solutions, such as atomic absorption spectrometry [34], inductively coupled plasma-optical emission spectrometry (ICP-OES) [35], anodic stripping voltammetry [36], and ion chromatography [37]. However, analytical methods can not directly measures the metal ions, in particular at ultra-trace concentration, in aqueous systems due to the lack of sensitivity and selectivity of these methods. Therefore, an efficient separation procedure is urgently required prior to the determination of noble metals for sensitive, accurate and interference-free determination of noble metals [38].

Several analytical methods are introduced for separation of analytes including liquid-liquid extraction [39], ion-exchange [40], co-precipitation [41], cloud-point extraction [42] and solid-phase extraction (SPE) [43]. SPE is considered to be one of the most powerful methods because it minimized the solvent usage and exposure, disposal costs, and extraction time for sample preparation. Several adsorbents have appeared due to the popularity of SPE for selective extraction of analytes, such as alumina [44], C18 [45], molecular imprinted polymers, cellulose [46], silica-gel [47,48], activated carbon [49,50] and carbon nanotubes [51,52]. This study was also planned to perform the analytical efficiency of CuO-TiO_2_ NSs phase as adsorbent on the selectivity and adsorption capacity of Fe(III) prior to its determination by ICP-OES. The selectivity of CuO-TiO_2_ NSs towards different metal ions, including Au(III), Cd(II), Co(II), Cr(III), Fe(III), Pd(II), and Zn(II), was performed in order to study the effectiveness of CuO- TiO_2_ NSs on the adsorption of selected metal ions. Here the calcined CuO-TiO_2_ NSs have significant properties such as large-surface area (surface-to-volume ratio) and static adsorption capacity. These offered high adsorbent features that enhanced the direct solid-phase adsorption towards the target metal-ions for the selective detection of Fe(III) ions. Based on the selectivity study, it was found that the selectivity of nanosheet-phase was the most towards Fe(III). The static adsorption capacity for Fe(III) was also executed in this study. Results of adsorption isotherm are confirmed that the adsorption process is mainly monolayer adsorption onto surface-phase containing a finite number of adsorption sites. Adsorption data of Fe(III) are well fit with the Langmuir-classical adsorption isotherm.

### Experimental section

#### Materials and methods

The λ_max_ (364.7 nm) of calcined CuO-TiO_2_ NSs was evaluated with UV/visible spectroscopy (UVO-2960, LABOMED Inc.). FT-IR spectra were measured with a spectro-photometer (Spectrum-100 FT-IR) in the mid-IR range, which was purchased from Bruker, USA. The powder X-ray diffraction (XRD) prototypes were measured with X-ray diffractometer (Rigaku X-ray difractometer, Mini-Flex 2) equipped with Cu-K_α_1 radiation (*λ* = 1.5406 nm) using a generator voltage (40.0 kV) and a generator current (35.0 mA). Morphology of CuO-TiO_2_ NSs was investigated on FE-SEM instrument (FESEM; JSM-7600F, Japan). The XPS measurements were executed on a Thermo Scientific K-Alpha KA1066 spectrometer (Germany). Monochromatic AlKα x-ray radiation sources were used as excitation sources, where beam-spot size was kept in 300.0 μm. The spectra was recorded in the fixed analyzer transmission mode, where pass energy was kept at 200 eV. The scanning of the spectra was performed at lower pressures (<10^−8^ Torr). 1000.0 mgL^−1^ stock standard solution of each Au(III), Cd(II), Co(II), Cr(III), Fe(III), Pd(II), and Zn(II) were purchased from Sigma-Aldrich (Milwaukee, WI, USA). All reagents were used of analytical and spectral purity grade. Doubly distilled de-ionized water was also used throughout the experimental studies. Analytical grade of copper chloride, titanium dioxide, and sodium hydroxide was used and purchased from Sigma-Aldrich Company. ICP-OES measurements were acquired by use of a Perkin Elmer ICP-OES model Optima 4100 DV, USA. The ICP-OES instrument was optimized daily before measurement and operated as recommended by the manufacturers. The ICP-OES spectrometer was used with following parameters: FR power (1300 kW), frequency (27.12 MHz), demountable quartz torch (Ar/Ar/Ar), plasma gas (Ar) flow (15.0 Lmin^−1^), auxiliary gas (Ar) flow (0.2 Lmin^−1^), nebulizer gas (Ar) flow (0.8 Lmin^−1^), nebulizer pressure (2.4 bar), glass spray chamber according to Scott (Ryton), sample pump flow rate (1.5 mLmin^−1^), integration time (3.0 s), replicates (3), and wavelength range of monochromator (165–460 nm). Selected metal ions were measured at wavelengths of 267.60 nm for Au(III), 228.80 nm for Cd(II), 238.90 nm for Co(II), 267.72 nm for Cr(III), 259.94 nm for Fe(III), 340.46 nm for Pd(II), and 206.20 nm for Zn(II).

#### Samples preparation and detection procedure

Stock solutions of Au(III), Cd(II), Co(II), Cr(III), Fe(III), Pd(II) and Zn(II) were prepared in 18.2 MΩ · cm distilled deionized water and stored in the dark at 4°C. For selectivity study, standard solutions (1.0 mgL^-1^) of each metal ion were prepared and adjusted to pH value of 5.0 with acetate buffer. Then, each standard solution was individually mixed with 25.0 mg CuO-TiO_2_ NSs. In this study, a fixed pH value of 5.0 was chosen for all metal ions in order to avoid any precipitation of other species, in particular for Fe(III). For example, Fe(III) usually forms a precipitation of Fe(OH)_3_ with buffer solutions at pH value greater than 5.0. For the investigation of Fe(III) static adsorption capacity, the standard solutions of 0, 5.0, 10.0, 15.0, 20.0, 25.0, 30.0, 50.0, 75.0, 125.0, and 150.0 mgL^−1^ were prepared and adjusted to the optimum pH value of 5.0 and individually mixed with 25.0 mg CuO-TiO_2_ NSs. All mixtures were mechanically shaken for 1 hr at room temperature.

### Synthesis of CuO-TiO_2_ nanosheets by a wet-chemical process

The liquid-phase precipitation was applied to prepare CuO-TiO_2_ NSs by a wet-chemical method from CuCl_2_ (0.1 M, 1.7048 g, 100.0 ml) and titanium dioxide (0.1 M, 0.799 g, 100.0 ml) as the precipitating agent in basic medium (pH ~ 10.5). The starting materials are put in de-ionized water to make 0.1M solution separately in round conical flask at room conditions. After addition of reducing agent (NaOH, adjusting pH at 11.0) into the reactant mixtures (CuCl_2_ & TiO_2_), it was stirred gradually for 12 hours and placed on a hot-plate (at 150.0°C, active solution temperature ~92°C). The starting materials (CuCl_2_, TiO_2_, and NaOH) were used without further purification for precipitation technique. Then the solution was washed with acetone and water consecutively and kept for drying at room conditions. The as-grown powder powders were calcined at 450.0°C in muffle furnace for 5 hours. Finally, the calcined products were characterized in features of their structural, morphological, and optical properties as well as applied for the detection of metal ions uptake.

## Results and discussion

### Optical characterization of CuO-TiO_2_ nanosheets

**Figure 1:**
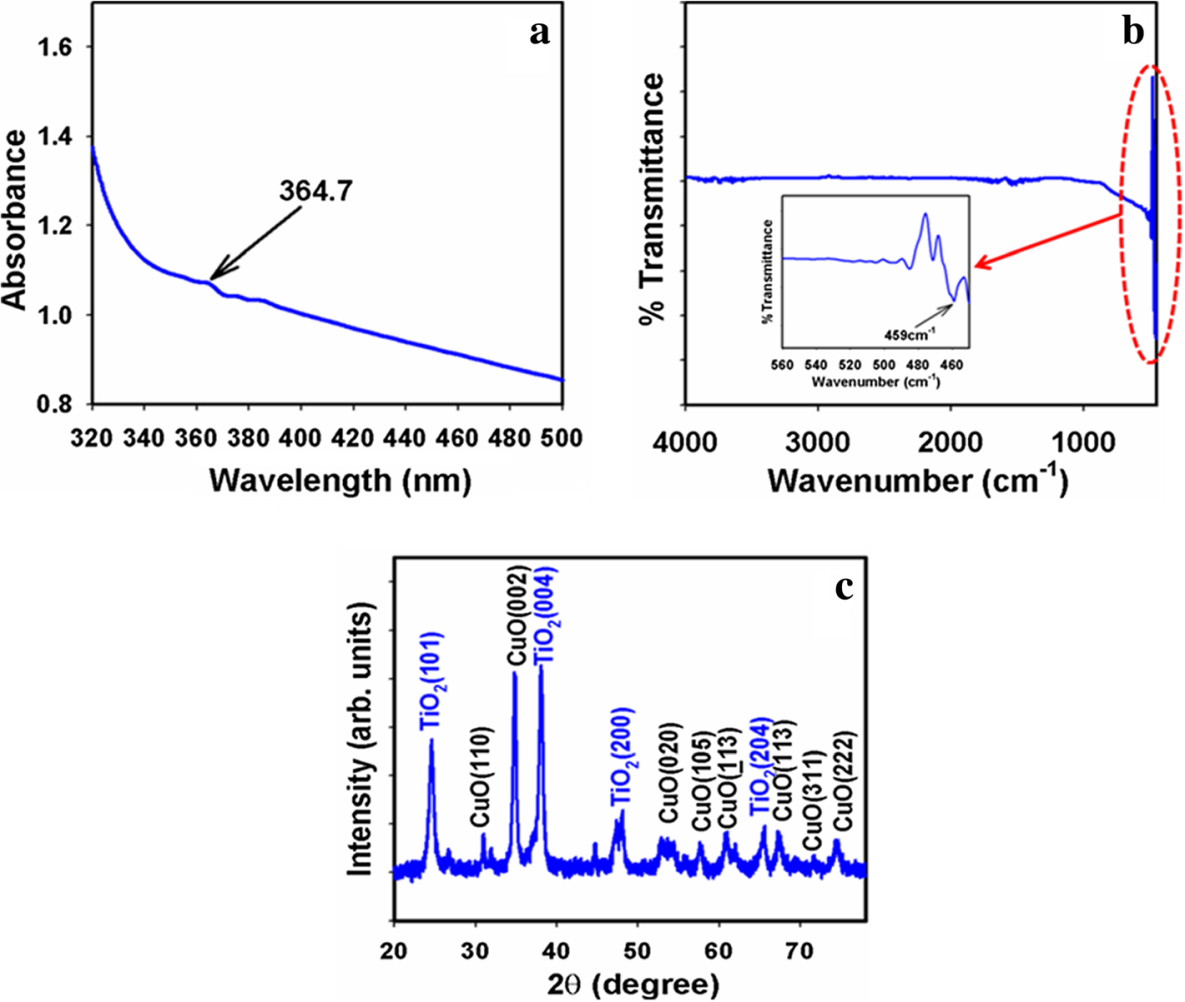
**(a) UV/visible spectroscopy and (b) FT-IR spectroscopy, and (c) powder X-ray diffraction pattern of calcined CuO-TiO**_**2 **_**nanosheets materials.**

The optical property of the calcined CuO-TiO_2_ NSs structure is one of the most important features for the evaluation of their photo-catalytic activity. The optical absorption spectra of NSs are measured by UV-visible spectrophotometer in the visible range (250.0 to 800.0 nm). In UV/visible absorption method, the outer-electrons of atoms or molecules are absorbed by incident radiation sources, which undergo electron transition from lower to higher energy levels. According to the phenomenon, the spectrum is obtained due to the optical absorption, which can be used to analyze the band-gap energy of CuO-TiO_2_ NSs. The optical absorption measurement was carried out at ambient conditions. From the absorption spectrum, an absorbance maximum is measured using NSs at ~364.7 nm, which is presented in Figure 1a. Band-gap energy (E_bg_) is calculated on the basis of the maximum absorption band of NSs and found to be 3.404 eV, according to following equation (i).

(i) $$E_{\mathrm{bg}}=\frac{1240}{\lambda }\left(\mathrm{eV}\right)$$

Where E_bg_ is the band-gap energy and λ_max_ is the wavelength (~364.7 nm) of the nanosheets. No extra peaks associated with impurities and structural defects were observed in the spectrums, which proved that the synthesized NSs control crystallinity of CuO-TiO_2_ NSs [53].

The CuO-TiO_2_ nanosheets were studied in term of atomic and molecular vibrations. FT-IR spectra basically in the region of 400–4000 cm^-1^ are executed at room conditions. Figure 1b displays the FT-IR spectrum of the calcined NSs structures. It represents band at 459 cm^-1^. This observed wide vibrational band (at 459 cm^-1^) could be assigned as a metal-oxygen (Cu-O & Ti-O) stretching vibration [54], which is demonstrated the configuration of doped nanomaterials. At low-frequency region, the vibrational bands are indicated the formation of CuO-TiO_2_ nanosheets. To confirm the crystal phases and crystallinity of the calcined CuO-TiO_2_ nanosheets, XRD pattern was employed and presented in Figure 1c. The obtained diffraction pattern is well matched with the base centered monoclinic CuO form. According to the JCPDS cards (45–0937), the lattice constants for calcined CuO NSs are found to be a = 4.6853 Å, b = 3.4257 Å, and c = 5.1303 Å. The phases are found the major characteristic peaks (indicated with black-color) with indices for calcined crystalline CuO at 2θ values of (110), (002), (020), (105), (113), (113), (311), and (222), which is presented in Figure 1c. The high-intensity of diffraction peaks in the obtained pattern clearly confirmed that the doped products are well-crystalline [55]. The CuO-TiO_2_ NSs are showed the crystalline nature of TiO_2_ peaks lying at 2θ = 25.3° (101), 2θ = 38.1° (004), 2θ = 48.6° (200), and 2θ = 66.1(204). The preferred orientation related to the plane (004) is observed the highest magnitude. All the peaks in XRD patterns can be indexed as crystalline phases of TiO_2_ and the diffraction data were in good agreement with JCPDS files # 021–1272 [56,57]. Crystallite size was calculated by Debye-Scherrer’s formula given by equation (ii)

(ii) $$D=\;K\lambda /\left(\mathrm{\beta cos}\;\theta \right)$$

Where D is the crystal size; λ is the wavelength of the X-ray radiation (λ = 0.15406 nm) for CuKα; K is usually taken as 0.9; and β is the line width at half-maximum height (FWHM) [58]. The average diameter and thickness of CuO-TiO_2_ nanosheets is close to ~1.07 μm and ~21 ± 5.0 nm respectively.

**Figure 2:**
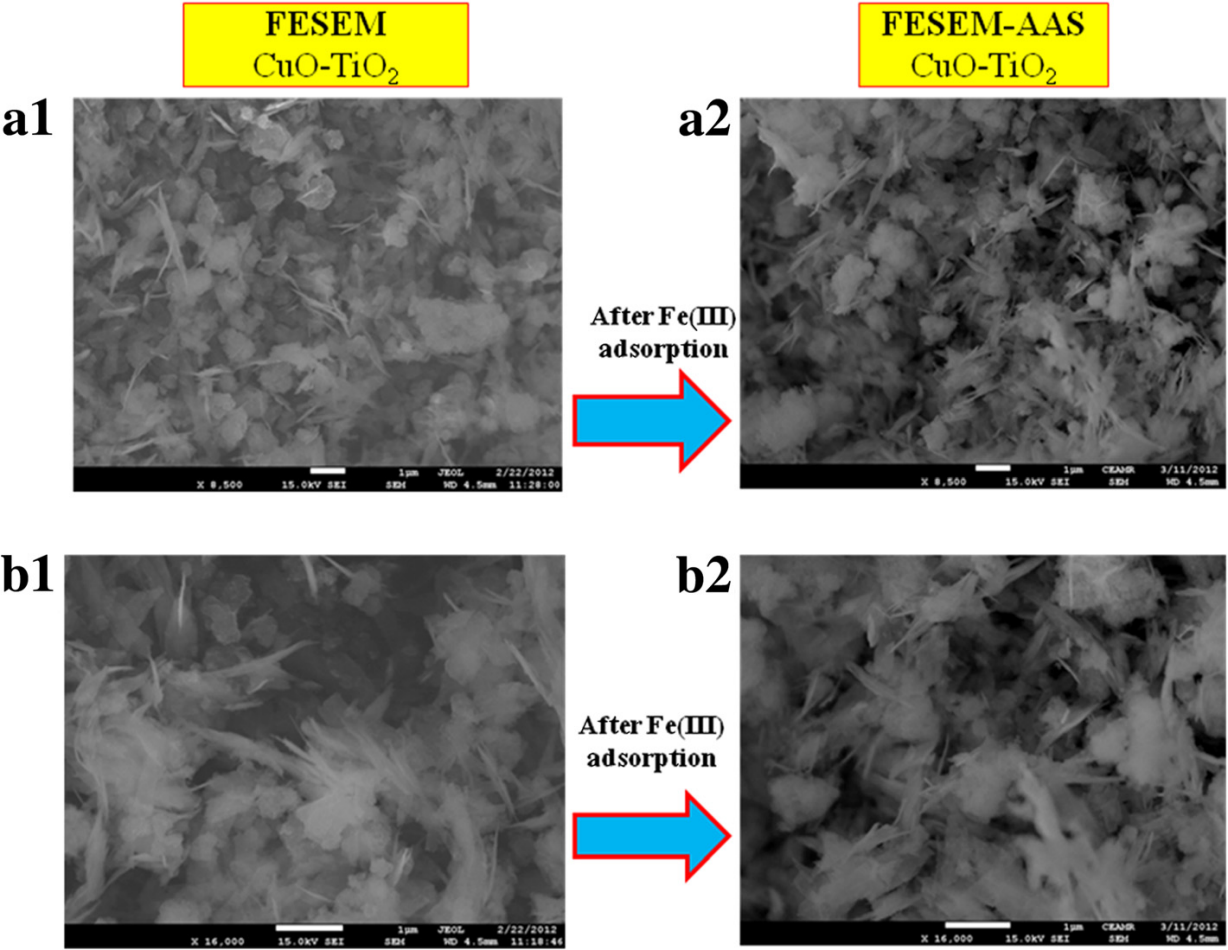
**Schematic representation and FESEM images of (a) before and (b) after Fe(III) adsorption on CuO-TiO**_**2 **_**NSs.**

High resolution FE-SEM images of calcined CuO-TiO_2_ NSs are presented in Figure 2a to Figure 2b. Low to high-magnified FE-SEM images [Figure 2(a1) to Figure 2(b1)] of aggregated nanosheets composed microstructure materials are displayed in sheet-shape. Microstructures are composed with nano-dimensional thickness of CuO-TiO_2_ sheets. The average length of NSs is calculated in the range of 1.0 μm to 1.3 μnm, which is close to ~1.07 ±10.0 μm. It is clearly exhibited from FE-SEM that the synthesized NS products are microstructure in regular sheet-shape with high-density of CuO-TiO_2_. The average cross-sectional thickness of sheets is measured from the FESEM image, which is close to ~21.0 ± 5.0 nm. It is interesting to note that most of the calcined CuO-TiO_2_ NSs are uniformly grown and possessing uniform sheet-shapes. In addition, the nano-dimensional sheets are perpendicularly arranged and aligned on the upper-portion of the calcined nanosheets. It is also proposed that the wet-chemically prepared nanostructures are composed with aggregated nanosheet composed microstructure of CuO-TiO_2_ NSs [59].

**Figure 3:**
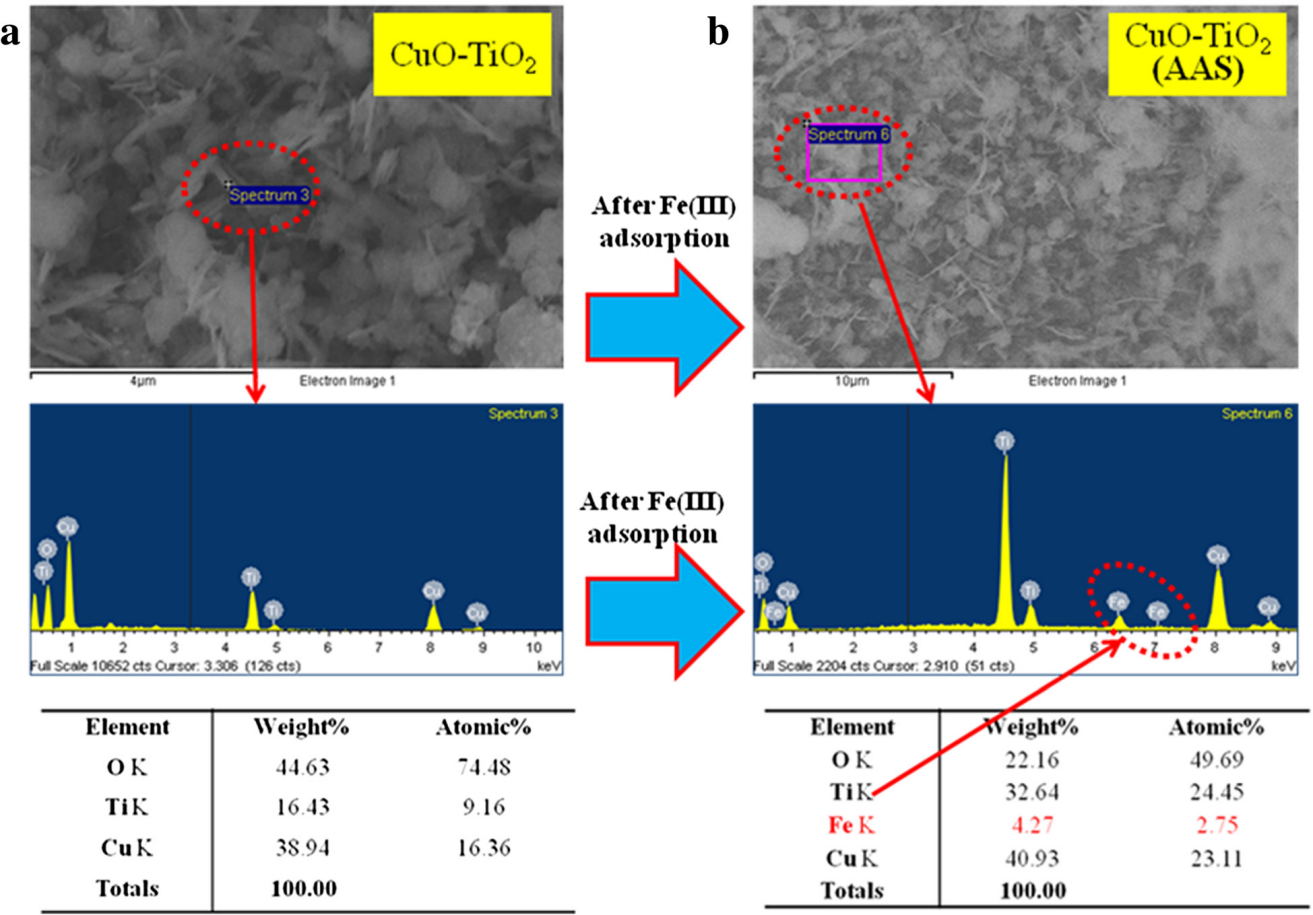
**EDS investigation of (a) before and (b) after iron(III) adsorption onto calcined CuO-TiO**_**2 **_**Nanosheets.**

The electron dispersive x-ray spectroscopy (EDS) analysis of CuO-TiO_2_ NSs are indicated the presence of copper (Cu), titanium (Ti), and oxygen (O) composition in the pure calcined NSs before metal ions uptake [Figure 3(a1 & b1)]. It is clearly displayed that the synthesized nanomaterial is contained only Cu, Ti, and O elements, which is presented in Figure 3(a1 & b1). No other peak related with any impurity has been detected in the FE-SEM coupled EDS, which confirms that the nanostructures are composed only with Cu (38.94%), Ti(16.43%), and O (44.63%), which is revealed in elemental analysis of Figure 3(b1) [area selected from Figure 3(a1)]. The incorporation of iron-ion onto the CuO-TiO_2_ NSs via adsorption (i.e., physi-sorption or chemi-sorption) method is confirmed. From the EDS observation, it is demonstrated that the iron ions are adsorbed onto the NSs, which is presented in Figure 3(a2) to Figure 3(b2). The elemental composition is measured via EDS analysis of Cu(40.93%), Ti(32.64%), O(22.16%), and Fe(4.27%), which is presented as a table with the Figures 3.

**Figure 4:**
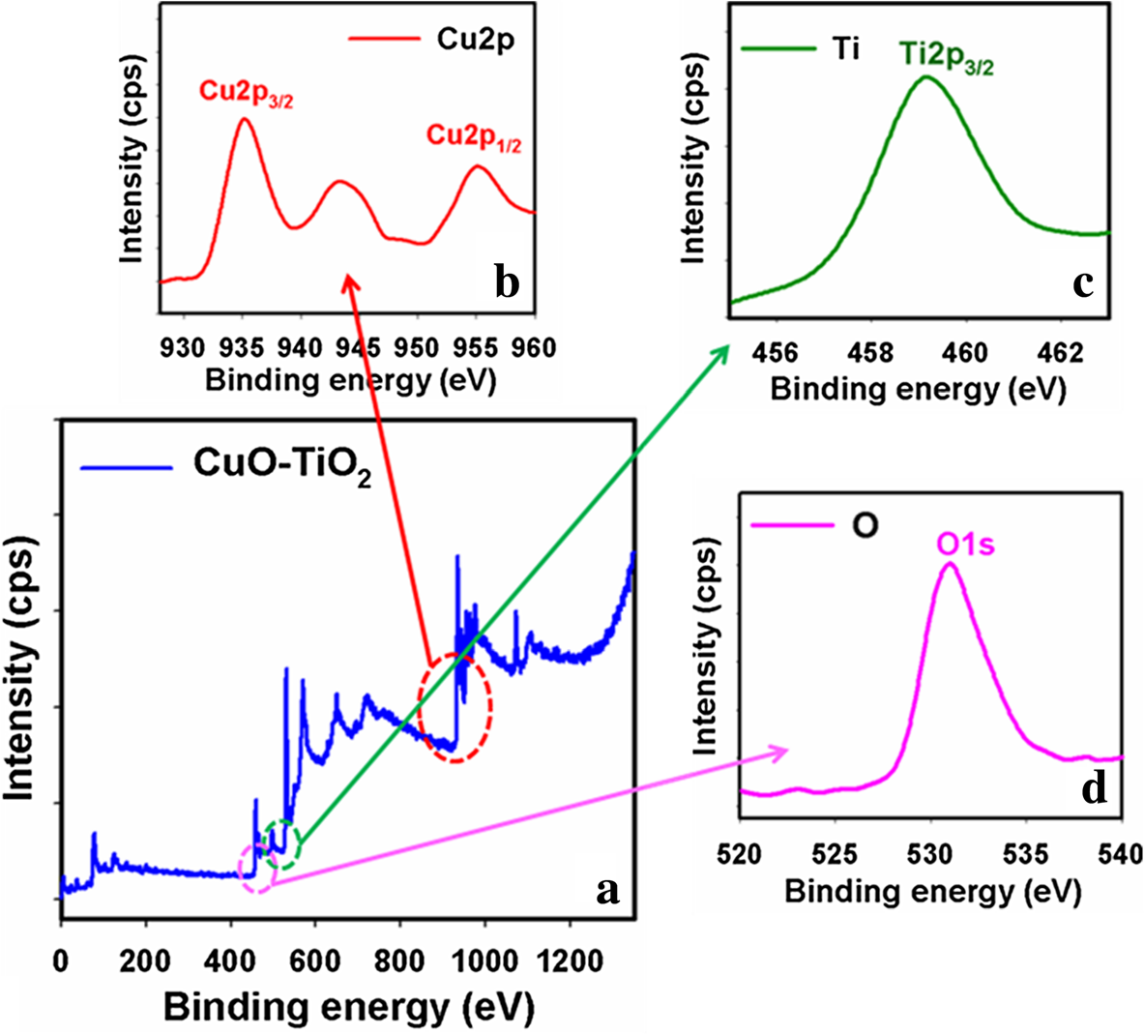
**XPS of (a) CuO-TiO**_**2 **_**NSs, (b) Cu2p level, (c) Ti2p level, and (d) O1s level acquired with MgKα radiation.**

X-ray photoelectron spectroscopy (XPS) is a quantitative spectroscopic method that determined the elemental-composition, kinetic energy, empirical-formula, chemical-state, binding energy, and electronic-state of the elements that present within a material. XPS spectra are acquired by irradiating a material with a beam of X-rays, while simultaneously determining the kinetic energy and number of electrons that get-away from the top one to ten nm of the material being analyzed. Here, XPS measurements were executed for CuO-TiO_2_ nanosheets to investigate the chemical states of CuO and TiO_2_. The XPS spectra of Cu2p and O1s are presented in Figure 4a. In Figure 4b, the spin orbit peaks of the Cu2p_(3/2)_ and Cu2p_(1/2)_ binding energy for all the samples appeared at around 935.2 eV and 956.1 eV respectively, which is in good agreement with the reference data for CuO [60]. In Figure 4c, the spin orbit peaks of the Ti2p_(3/2)_ binding energy for codoped sample appeared around at 559.5 eV, which is in good agreement with the reference data for TiO_2_[61]. The O1s spectrum shows a peak at 531.9 eV in Figure 4d. The peak at 531.9 eV is assigned to lattice oxygen, may be indicated to oxygen (ie, O_2_^-^) presence in the CuO-TiO_2_ NSs [62]. XPS compositional analyses investigated the co-existence of the two single-phase of CuO and TiO_2_ materials. Therefore, it is concluded that the wet-chemically prepared CuO-TiO_2_ materials have NSs phase contained two different materials. Also, this conclusion is reliable with the XRD data significantly.

### Selectivity study of CuO-TiO_2_ nanosheets

Selectivity of newly introduced CuO-TiO_2_ NSs phase towards various metal ions was measured based on determination of the distribution coefficient of NSs phases. The distribution coefficient (*K*_*d*_) can be obtained from the following equation (iii) [63]:

(iii) $$K_{d}=\left(C_{o}–C_{e}/C_{e}\right)\times \left(V/m\right)$$

where *C*_*o*_ and *C*_*e*_ refer to the initial and final concentrations before and after filtration with CuO-TiO_2_ NSs, respectively, *V* is the volume (mL) and *m* is the weight of CuO-TiO_2_ NSs phase (g). Distribution coefficient values of all metal ions investigated in this study are reported in Table 1. It can be clearly observed from the Table 1 that the greatest distribution coefficient value was obtained for Fe(III) with CuO-TiO_2_ (50546.392 mLg^−1^) as compared to pure TiO_2_ adsorbent (3557.885 mLg^−1^). As shown in Table 1, the amount of Fe(III) is almost extracted by CuO-TiO_2_ phase. In addition, minimal to no change in the selectivity is observed for both CuO-TiO_2_ and TiO_2_ adsorbents toward other metal ions included in this study. Results of selectivity study provided that the newly synthesized CuO-TiO_2_ phase is the most selective towards Fe(III) among all metal ions. The highest selectivity of CuO-TiO_2_ adsorbent toward Fe(III) can be attributed to additional incorporated oxygen atoms _(_donor) presented in CuO-TiO_2_ phase, as a result of modification of TiO_2_ with CuO. Thus, both incorporated oxygen donor atoms presented in TiO_2_ and CuO-TiO_2_ phases are able to selectively bind with Fe(III) through an electrostatic attraction or a chelating mechanism.

**Table 1:** **Selectivity study of CuO-TiO**_**2**_**NSs phase adsorption towards different metal ions at pH 5.0 and 25°C (*N*** **= 5)**

**Metal Ion**	***q***_***e***_***(mgg***^***−1***^***)*, TiO**_**2**_	***q***_***e***_***(mgg***^***−1***^***)*, CuO-TiO**_**2**_	***K***_***d***_**(mLg**^**−1**^**)*,*TiO**_**2**_	***K***_***d***_**(mLg**^**−1**^**)*,*CuO-TiO**_**2**_
**Fe(III)**	**0.781**	**0.981**	**3557.885**	**50546.392**
**Au(III)**	0.150	0.153	175.938	180.638
**Zn(II)**	0.054	0.060	57.082	63.830
**Pd(II)**	0.047	0.050	49.318	52.632
**Co(II)**	0.036	0.040	37.344	41.667
**Cd(II)**	0.007	0.010	7.049	10.101
**Cr(III)**	0.009	0.010	9.082	10.101

### Static adsorption capacity

For determination of the static uptake capacity of Fe(III) on CuO-TiO_2_ NSs phase, 25.0 mL of Fe(III) sample solutions with different concentrations (0–150.0 mgL^−1^) were adjusted to pH 5.0 and individually mixed with 25.0 mg of CuO-TiO_2_ NSs. These mixtures were mechanically shaken for a hr at room temperature. Static adsorption capacity was obtained using equation (iv) as follows:

(iv) $$q_{e}=\frac{\left(C_{o}-C_{e}\right)V}{m}$$

**Figure 5:**
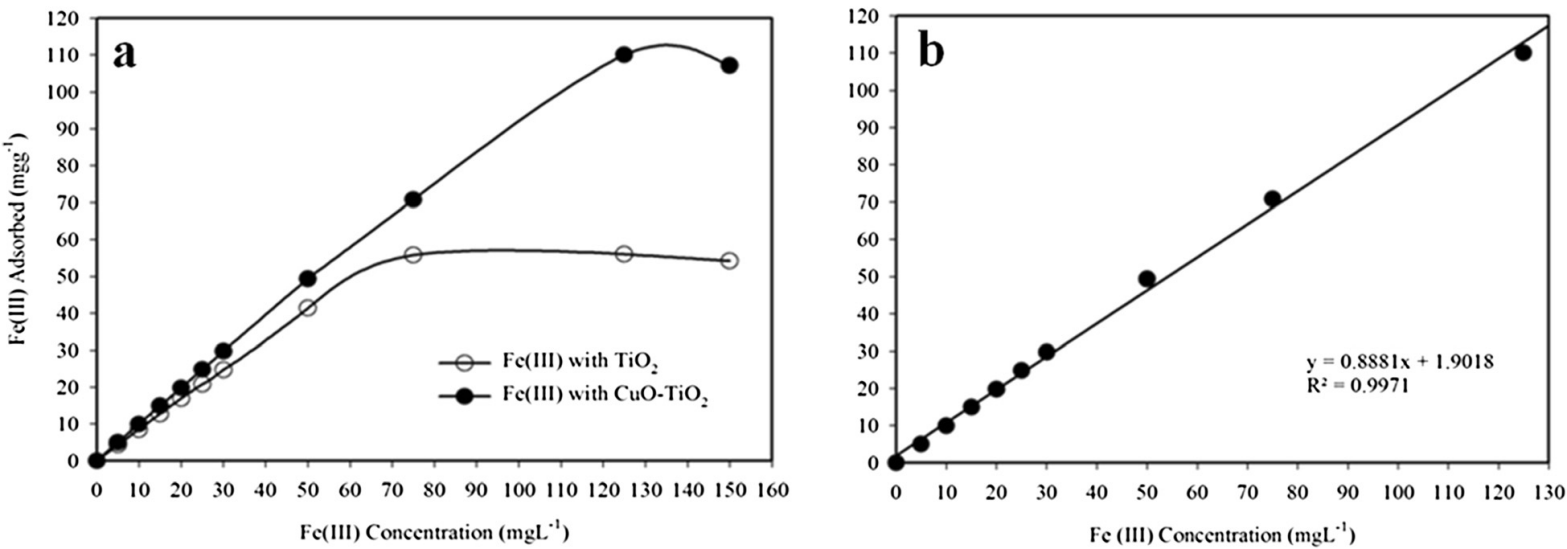
**(a) Adsorption profile and (b) sensitivity of Fe(III) on 25.0 mg CuO-TiO**_**2 **_**NSs phase in relation to the concentration at pH 5.0 and 25°C.**

Where *q*_e_ represents the adsorbed Fe(III) by the CuO-TiO_2_ NSs phase (mgg^−1^), C_ο_ and C_e_ are the initial and equilibrium concentrations of Fe(III) ion in solution (mgL^−1^), respectively, *V* is the volume (L) and *m* is the weight of NSs phase (g). Figure 5 shows the static adsorption capacity of NSs for Fe(III) obtained from the experiment of adsorption isotherm. In this study, the adsorption capacity of NSs was determined for Fe(III), which is close to be 110.06 mgg^−1^. This is the higher adsorption capacity value obtained, which is compared with previously reports for Fe(III) in various studies such as 7.00 [64], 18.30 [65], 28.69 [66], 28.90 [67], and 173.14 [68] mgg^−1^. Moreover, under the same batch conditions, the adsorption capacity of Fe(III) on pure TiO_2_ and doped CuO-TiO_2_ NSs adsorbent is estimated to be 56.06 mgg^−1^ and 110.06 mgg^−1^ respectively, which is presented in Figure 5. From this comparative study, it is concluded that the adsorption capacity for Fe(III) was improved by 96.33% with the newly synthesized CuO-TiO_2_ NSs adsorbent as compared to pure TiO_2_ phase. The sensitivity (slope) and linearity (R^2^) of Fe(III) ion using CuO-TiO_2_ NSs phase is calculated from the calibration plot, which is close to 0.8881 Lg^-1^ and 0.9971 respectively.

### Adsorption isotherm models

Experimental equilibrium adsorption data were analyzed using different models in order to develop an equation that accurately represents the obtained results. Langmuir equation is based on an assumption of a monolayer adsorption onto a completely homogeneous surface with a finite number of identical sites and a negligible interaction between the adsorbed molecules. The Langmuir adsorption isotherm model is governed by the following relation (v) [69]:

(v) $$C_{e}/q_{e}=\left(C_{e}/Q_{o}\right)+1/Q_{o}b$$

Where *C*_e_ corresponds to the equilibrium concentrations of Fe(III) ion in solution (mgmL^−1^) and *q*_e_ is the adsorbed metal ion by the adsorbate (mgg^−1^). The symbols *Q*_o_ and *b* refer to Langmuir constants related to adsorption capacity (mgg^−1^) and energy of adsorption (Lmg^−1^), respectively. These constants can be determined from a linear plot of *C*_e_/*q*_e_ against *C*_e_ with a slope and intercept equal to 1/*Q*_o_ and 1/*Q*_o_*b*, respectively. Moreover, the essential characteristics of Langmuir-adsorption isotherm can be represented in terms of a dimension-less constant separation factor or equilibrium parameter, *R*_*L*_, which is defined as *R*_*L*_ = 1/(1 + *bC*_o_), where *b* is the Langmuir constant (indicates the nature of adsorption and the shape of the isotherm); *C*_o_ the initial concentration of the analyte. The *R*_*L*_ value indicates the type of the isotherm, and *R*_*L*_ values between 0 and 1 represent a favorable adsorption [70].

**Figure 6:**
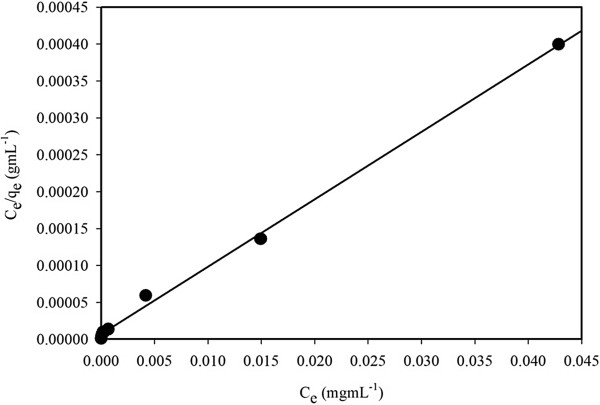
**Langmuir adsorption isotherm model of Fe(III) adsorption on 25 mg CuO-TiO**_**2 **_**NSs phase at pH 5.0 and 25°C.** Adsorption experiments were obtained at different concentrations (0–150 mgL^−1^) of Fe(III) under static conditions.

The experimental isotherm data were fit-well with the Langmuir equation based on the least square fit, as shown in Figure 6, supporting the validity of Langmuir adsorption isotherm model for the adsorption process. Consequently, adsorption isotherm data was indicated that the adsorption process was mainly monolayer on a homogeneous adsorbent surface of NSs. Langmuir constants *Q*_o_ and *b* are found to be 109.46 mgg^−1^ and 1.30 Lmg^-1^, respectively. The correlation coefficient obtained from the Langmuir model is found to be *R*^*2*^ = 0.998 for adsorption of Fe(III) on CuO-TiO_2_ NSs. Furthermore, the static adsorption capacity (109.46 mgg^−1^) calculated from Langmuir equation was in agreement with that (110.06 mgg^−1^) of the experimental isotherm study. The *R*_*L*_ value of Fe(III) adsorption on the NSs is 0.01, indicating a highly favorable adsorption process based on the Langmuir classical adsorption isotherm model.

## Conclusions

The calcined CuO-doped TiO_2_ NSs are successfully prepared by a wet-chemical technique combined with a heat-treatment and characterized in detail in terms of their morphological, structural, and optical properties. It is displayed that the synthesized microstructures are possessed monoclinic structure having good optical properties. The static-uptake capacity of the NSs phase for selective adsorption and determination of Fe(III) in aqueous solution was investigated. Reasonable static-uptake capacity of 110.06 mgg^−1^ with NSs adsorbent for Fe(III) in aqueous solution was achieved. Adsorption data of Fe(III) was well-fit with the Langmuir adsorption isotherm model. Thus, the method may show considerable promise for using it as an effective approach for a selective separation and determination of Fe(III) in complex matrices. The obtained nanosheets composed CuO-TiO_2_ microstructures is a promising candidate for potential application in metal ions uptake.

## Competing interests

The authors declare that they have no competing interests.

## Authors’ contributions

MMR made a significant contribution to preparation and characterization of nanomaterials, survey results, and data collection and their analysis and writing the manuscript. SBK participated in the synthesis of samples and collection of data and experimental work. HMM carried out the application part on “Metal ion uptake using doped nanomaterials” AMA has revised the manuscript for intellectual content. KAA participated in the UV/vis. and FTIR characterization of samples and collection of data and plotted the graph. All authors read and approved the final manuscript.
